# Supplementary material for: The Clinical and Economic Impact of Antibiotic Resistance in China: A Systematic Review and Meta-Analysis
Source: Antibiotics (Basel). 2019 Aug 10;8(3):115. doi: 10.3390/antibiotics8030115 (PMC6784351; doi:10.3390/antibiotics8030115)
Supplement: Supplementary file 1 [file antibiotics-08-00115-s001.pdf]

Supplementary data 1. Search terms and search strategies

1. PubMed [5063]
(antimicrobial* [Title/Abstract] OR antibiotic [Title/Abstract] OR microbial [Title/Abstract] OR bacterial*[Title/Abstract] OR multi-drug [Title/Abstract] OR multidrug [Title/Abstract] OR multiple-drug[Title/Abstract] OR multiple drug [Title/Abstract] OR gram-positive[Title/Abstract] OR gram-negative[Title/Abstract] OR enterococcus[Title/Abstract] OR escherichia[Title/Abstract] OR klebsiella[Title/Abstract] OR enterobacter[Title/Abstract] OR pseudomonas[Title/Abstract] OR acinetobacter[Title/Abstract] OR staphylococcus[Title/Abstract]) **AND**
(resistan*[Title/Abstract] OR MDR[Title/Abstract] OR susceptib*[Title/Abstract] OR nonsusceptib*[Title/Abstract]) **AND**
(economic*[Title] OR cost*[Title] OR length of stay[Title] OR hospital stay*[Title] OR mortality[Title] OR clinical[Title] OR resource utilization[Title] OR burden[Title] OR outcome[Title]) **AND**
(inpatients [Mesh terms] OR outpatients [Mesh terms] OR inpatient*[Title/Abstract] OR outpatient*[Title/Abstract] OR hospital[Title/Abstract] OR hospitals[Title/Abstract] OR hospitalization[Title/Abstract] OR hospitalized[Title/Abstract])

2. Web of Science [1250]
(antimicrobial* OR antibiotic OR microbial OR bacterial* OR multi-drug OR multidrug OR multiple-drug OR multiple drug OR gram-positive OR gram-negative OR enterococcus OR escherichia OR klebsiella OR enterobacter OR pseudomonas OR acinetobacter OR staphylococcus) [Topic] **AND**
(resistan* OR MDR OR susceptib* OR nonsusceptib*) [Topic] **AND**
(inpatients OR outpatients OR patients OR patient OR hospital OR hospitals OR hospitalization OR hospitalized) [Topic] **AND**
(economic* OR cost* OR length of stay OR hospital stay* OR mortality OR clinical OR outcome OR burden OR resource utilization) [Title]

3. Embase [7010]
(‘antimicrobial*’:ab,ti OR ‘antibiotic’:ab,ti OR ‘microbial’:ab,ti OR ‘bacterial’:ab,ti OR ‘multi-drug’:ab,ti OR ‘multidrug’:ab,ti OR ‘multiple-drug’:ab,ti OR ‘multiple drug’:ab,ti OR ‘gram-positive’:ab,ti OR ‘gram-negative’:ab,ti OR ‘enterococcus’:ab,ti OR ‘escherichia’:ab,ti OR ‘klebsiella’:ab,ti OR ‘enterobacter’:ab,ti OR ‘pseudomonas’:ab,ti OR ‘acinetobacter’:ab,ti OR ‘staphylococcus’:ab,ti) **AND**
(‘resistan*’:ab,ti OR ‘MDR’:ab,ti OR ‘susceptib*’:ab,ti OR ‘nonsusceptib*’:ab,ti) **AND**
(‘economic*’:ti OR ‘cost*’:ti OR ‘length of stay’:ti OR ‘hospital stay*’:ti OR ‘mortality’:ti OR ‘clinical’:ti OR ‘outcome’:ti OR ‘burden’:ti OR ‘resource utilization’:ti) **AND**
(‘hospital patient’/exp OR ‘inpatients’:ab,ti OR ‘outpatients’:ab,ti OR ‘hospital’:ab,ti OR ‘hospitals’:ab,ti OR ‘hospitalization’:ab,ti OR ‘hospitalized’:ab,ti)

4. CNKI [242]
SU=(’抗生素’+’抗菌药物+’病原菌’+’致病菌’+’抗菌素’+’金黄色葡萄球菌’+’金葡菌’+’肠球菌’+’大肠埃希*’+’大肠杆菌’+’肠杆菌’+’肺炎克雷*’+’铜绿假单胞菌’+’鲍曼不动杆菌’+’鲍氏不动杆菌’+’革兰阴性菌’+’革兰氏阴性*’+’革兰阳性菌’+’革兰氏阳性*’) AND SU=(’耐药’+’敏感’+’多重耐药’+’多药耐药’) AND SU=(‘住院费用’+’医疗费用’+’经济负担’+’疾病负担’+’住院天数’+’住院时间’+’住院日’+’死亡率’+’病死率’) AND SU=(‘患者’+’病人’)

5. Wanfang data [105]
主题:(抗生素+抗菌药物+病原菌+致病菌+抗菌素+金黄色葡萄球菌+金葡菌+肠球菌+”大肠埃希*”+肠杆菌+”肺炎克雷*”+铜绿假单胞菌+鲍曼不动杆菌+鲍氏不动杆菌+革兰阴性菌+革兰氏

阴性+革兰阳性菌+革兰氏阳性)*主题:(耐药+敏感+多重耐药+多药耐药)*题名或关键词:(住院费用+医疗费用+经济负担+疾病负担+住院天数+住院时间+住院日+死亡率+病死率)*主题:(患者+病人)

6. CQVIP [23]
(K=(抗生素 OR 抗菌药物 OR 病原菌 OR 致病菌 OR 抗菌素 OR 金黄色葡萄球菌 OR 金葡菌 OR 肠球菌 OR 大肠埃希 OR 肠杆菌 OR 肺炎克雷 OR 铜绿假单胞菌 OR 鲍曼不动杆菌 OR 鲍氏不动杆菌 OR 革兰阴性菌 OR 革兰氏阴性 OR 革兰阳性菌 OR 革兰氏阳性)) AND (K=(耐药 OR 敏感 OR 多重耐药 OR 多药耐药)) AND (K=(住院费用 OR 医疗费用 OR 经济负担 OR 疾病负担 OR 住院天数 OR 住院时间 OR 住院日 OR 死亡率 OR 病死率)) AND (K=(患者 OR 病人))

Supplementary data 2: Study characteristics

Table S1. Studies describing hospital mortality among inpatients with antibiotic resistance and multi-drug resistance.

Author	Year	Type of study	Methods	Province	Hospital setting	Study period	Study population	Infection Type	Hospital wards	Bacteria
Guo et al. [43]	2017	retrospective cohort	PSM/ significant test	Shanghai	single	2008-2013	adult patients ≥16 years	HAI	N/A	*A. baumannii*
Hu et al. [42]	2014	retrospective	binary multivariate regression	Zhejiang	multiple	2011-2012	N/A	NI	N/A	gram-negative
Huang [41]	2015	retrospective	significant test	Zhejiang	single	2009-2011	N/A	HAI	ICU	*K. pneumoniae*
Li et al. [38]	2014	retrospective case-control	significant test	Guangdong	single	2011	N/A	N/A	ICU	gram-negative/gram positive
Liang [36]	2014	prospective	multivariate regression /respiratory/emergency	Hebei	single	2012-2013	patients with mechanical ventilation	pulmonary HAI	ICU/ respiratory/ emergency	*A. baumannii*
Lv et al. [34]	2015	retrospective	significant test	Zhejiang	single	2011-2014	N/A	infection	N/A	*P. aeruginosa*
Pei [32]	2015	retrospective	significant test	Anhui	single	2010-2012	N/A	infection	N/A	*A. baumannii*
Wang [31]	2018	retrospective	significant test	Zhejiang	single	2016-2017	N/A	BSI	N/A	gram-negative
Wang et al. [29]	2016	retrospective case-control	multivariate logistic regression	Jiangsu	single	2010-2014	N/A	pneumonia	N/A	*A. baumannii*
Zhang et al. [24]	2013	retrospective case-control	matching/ significant test	Hubei	single	2009-2011	N/A	BSI	N/A	*S. aureus*
Zhou et al. [23]	2015	retrospective case-control	multivariate logistic regression	Sichuan	single	2009-2013	N/A	BSI	N/A	*S. aureus*
Chen et al. [22]	2016	retrospective case-control	significant test PSM/ significant test	Jiangsu	single	2013-2014	adult patients ≥18 years	HAI pneumonia	N/A	*S. aureus*
Cui et al. [21]	2012	retrospective case-control	matching/multivariate logistic regression	Beijing	single	2007-2009	N/A	NI	N/A	*A. baumannii*
Long [20]	2015	retrospective case-control	matching/ significant test	Shanghai	single	2006-2014	children	BSI	N/A	Gram-negative
Zhu et al. [1]	2016	retrospective	multivariate logistic regression	Guangdong	single	2011-2014	patients≥1 year	nosocomial BSI	N/A	*S. aureus*
Yang et al. [3]	2018	retrospective case-control	cox regression analysis	Chongqing	single	2011-2015	adult inpatients≥ 18 years	nosocomial bacteremia	N/A	*A. baumannii*
Chen et al. [6]	2018	retrospective cohort	significant test PSM	Zhejiang	single	2014-2018	adult inpatients ≥18 years	N/A	N/A	*P. aeruginosa*
Meng et al. [7]	2017	retrospective case-control	multivariate logistic regression	Hunan	single	2012-2015	N/A	HAI	N/A	*E. coli*
Zheng et al. [8]	2013	retrospective cohort	multivariate backward logistic regression	Jiangsu	single	2006-2011	N/A	nosocomial pneumonia	N/A	*A. baumannii*
Yuan et al. [9]	2017	retrospective cohort	multiple stepwise logistic regression	Shanghai	single	2013	N/A	NI	N/A	*P. aeruginosa*
Xiao et al. [10]	2018	retrospective	binary logistic regression	Zhejiang	single	2013-2015	adult inpatients ≥16 years	BSI	N/A	*K. pneumoniae*
Wang et al. [11]	2018	retrospective case-control	multiple stepwise regression	Beijing	single	2010-2014	adult inpatients ≥ 18 years	infection	N/A	*K. pneumoniae*

Tian et al. [12]	2016	retrospective	multivariate logistic analysis	Shanghai	single	2011-2015	N/A	BSI	N/A	*K. pneumoniae*
Jiao et al. [13]	2015	retrospective case-control	multiple stepwise regression	Shanghai	single	2010-2011	adult inpatients ＞18 years	HAI or colonization	N/A	*K. pneumoniae*
Huang et al. [14]	2018	retrospective cohort	PSM/ significant test significant test	Sichuan	single	2017	N/A	N/A	N/A	*K. pneumoniae*
Yang et al. [19]	2009	retrospective cohort	multiple stepwise linear regression	Beijing/ Shandong/ Hubei/ Sichuan/ Ningxia	multiple	2005	N/A	N/A	N/A	gram positive/ gram negative
Cao et al. [16]	2004	retrospective case control/ cohort	multivariable logistic regression	Beijing	single	1999-2002	N/A	N/A	N/A	*P. aeruginosa*
Jia et al. [17]	2015	retrospective case control	significant test	Chongqing	single	2011-2014	N/A	N/A	N/A	*Enterococcus*
Cai et al. [18]	2012	retrospective	significant test	Hubei	single	2009-2011	Children	N/A	pediatric ICU	*A. baumannii*

PSM: propensity score matching; HAI: healthcare acquired infection; NI: nosocomial infection; BSI: bloodstream infection; ICU: intensive care unit; *A. baumannii*: *Acinetobacter baumannii*; *K. pneumoniae*: *Klebsiella pneumoniae*; *P. aeruginosa*: *Pseudomonas aeruginosa*; *S. aureus*: *Staphylococcus aureus*; *E. coli*: *Escherichia coli*.

Table S2. Studies describing hospital stay among patients with antibiotic resistance and multi-drug resistance.

Author	Year	Type of study	Methods	Province	Hospital setting	Study period	Study population	Infection Type	Hospital wards	Bacteria
Fu et al. [44]	2014	case-control	matching/ significant test	Fujian	single	2012-2013	N/A	infection	N/A	*S. aureus*
Guo et al. [43]	2017	retrospective cohort	PSM/ significant test	Shanghai	single	2008-2013	adult patients ≥16 years	HAI	N/A	*A. baumannii*
Hu et al. [42]	2014	retrospective	significant test	Zhejiang	multiple	2011-2012	N/A	NI	N/A	gram-negative
Huang [41]	2015	retrospective	significant test	Zhejiang	single	2009-2011	N/A	HAI	ICU	*K. pneumonia*
Jiang et al. [40]	2016	retrospective	matching/ significant test	Shandong	single	2015	N/A	NI	N/A	gram-negative/gram-positive
Li et al. [39]	2018	retrospective	significant test	Anhui	single	2016-2017	patients with bronchiectasis and infection	community-acquired infection	respiratory and ICU	gram-negative/gram-positive
Li et al. [37]	2016	retrospective	significant test	Fujian	single	2009-2013	N/A	NI	respiratory	K. pneumonia
Liang [36]	2014	prospective	significant test	Hebei	single	2012-2013	patients with mechanical ventilation	pulmonary HAI	ICU/ respiratory/ emergency	*A. baumannii*
Liu [35]	2018	retrospective case-control	PSM/ significant test	Hubei	multiple	2013-2015	N/A	N/A	N/A	gram-negative/gram-positive
Lv et al. [34]	2015	retrospective	significant test	Zhejiang	single	2011-2014	N/A	infection	N/A	*P. aeruginosa*
Pan et al. [33]	2018	retrospective	significant test	Yunnan	single	2012-2015	surgical inpatients	infection	N/A	gram-negative/gram-positive
Pei [32]	2015	retrospective	significant test	Anhui	single	2010-2012	N/A	infection	N/A	*A. baumannii*
Wang [31]	2018	retrospective	significant test	Zhejiang	single	2016-2017	N/A	BSI	N/A	gram-negative
Jiang et al. [30]	2018	retrospective	significant test	Sichuan	single	2014-2015	N/A	infection	respiratory medicine	gram-negative
Wang et al. [29]	2016	retrospective case-control	significant test	Jiangsu	single	2010-2014	N/A	pneumonia	N/A	*A. baumannii*

Wu et al. [28]	2018	retrospective case-control	matching/ significant test	Chongqing	single	2014-2016	N/A	infection	N/A	*A. baumannii*
Xing et al. [27]	2017	retrospective case-control	significant test	Shandong	single	2013-2015	N/A	infection	N/A	gram-negative/gram-positive
Xu et al. [26]	2017	retrospective	significant test	Hubei	single	2015	N/A	infection	N/A	*E. coli*<br>K. pneumonia<br>*Proteus mirabilis*<br>*A. baumannii*<br>*P. aeruginosa*<br>*Enterobacter cloacae*<br>*S. aureus*<br>*coagulase-negative Staphylococci*
Yu [25]	2016	retrospective case-control	significant test	Zhejiang	single	2013-2014	N/A	infection	N/A	*S. aureus*
Zhang et al. [24]	2013	retrospective case-control	matching/ significant test	Hubei	single	2009-2011	N/A	BSI	N/A	*S. aureus*
Zhou et al. [23]	2015	retrospective case-control	significant test	Sichuan	single	2009-2013	N/A	BSI	N/A	*S. aureus*
Chen et al. [22]	2016	retrospective case-control	significant test<br>PSM/ significant test<br>significant test<br>PSM/ significant test	Jiangsu	single	2013-2014	adult patients ≥18 years	HAI pneumonia	N/A	*S. aureus*
Cui et al. [21]	2012	retrospective case-control	matching/significant test	Beijing	single	2007-2009	N/A	NI	N/A	*A. baumannii*
Long [20]	2015	retrospective case-control	matching/ significant test	Shanghai	single	2006-2014	children	BSI	N/A	gram-negative
Zhu et al. [1]	2016	retrospective	significant test	Guangdong	single	2011-2014	patients≥1 year	nosocomial BSI	N/A	*S. aureus*
Hu et al. [2]	2010	retrospective	generalized linear model	Shanghai/ Beijing/ Zhejiang	multiple	2006-2007	N/A	IAI	N/A	*E. coli/ Klebsiella spp.*
Zhen et al. [4]	2017	retrospective	multivariate linear analysis	Zhejiang	single	2013-2015	N/A	N/A	N/A	*A. baumannii*
Zhen et al. [5]	2018	retrospective	significant test	Zhejiang	single	2013-2015	N/A	IAI	N/A	gram-negative/gram-positive
Chen et al. [6]	2018	retrospective cohort	significant test<br>PSM<br>significant test<br>PSM	Zhejiang	single	2014-2018	adult inpatients ≥18 years	N/A	N/A	*P. aeruginosa*
Wang et al. [11]	2018	retrospective case-control	significant test	Beijing	single	2010-2014	adult inpatients ≥ 18 years	infection	N/A	*K. pneumoniae*
Tian et al. [12]	2016	retrospective	significant test	Shanghai	single	2011-2015	N/A	BSI	N/A	*K. pneumoniae*
Jiao et al. [13]	2015	retrospective case-control	significant test	Shanghai	single	2010-2011	inpatients ＞18 years	HAI or colonization	N/A	*K. pneumoniae*

Huang et al. [14]	2018	retrospective cohort	PSM/ significant test	Sichuan	single	2017	N/A	N/A	N/A	*K. pneumoniae*
			significant test							
			significant test							
			significant test							
Yang et al. [19]	2009	retrospective cohort	multiple stepwise linear regression	Beijing/ Shandong/ Hubei/ Sichuan/ Ningxia	multiple	2005	N/A	N/A	N/A	gram-negative/gram-positive
Li et al. [15]	2016	retrospective	significant test	Guangdong	multiple	2008-2013	adult patients ≥18 years	N/A	N/A	*S. aureus*
Jia et al. [17]	2015	retrospective case control	significant test	Chongqing	single	2011-2014	N/A	N/A	N/A	*Enterococcus*
Cai et al. [18]	2012	retrospective	significant test	Hubei	single	2009-2011	Children	N/A	pediatric ICU	*A. baumannii*

PSM: propensity score matching; HAI: healthcare acquired infection; NI: nosocomial infection; BSI: bloodstream infection; IAI: intra-abdominal infection; ICU: intensive care unit; *A. baumannii*: *Acinetobacter baumannii*; *K. pneumoniae*: *Klebsiella pneumoniae*; *P. aeruginosa*: *Pseudomonas aeruginosa*; *S. aureus*: *Staphylococcus aureus*; *E. coli*: *Escherichia coli*.

Table S3. Studies describing hospital costs among patients with antibiotic resistance and multi-drug resistance.

Author	Year	Type of study	Methods	Province	Hospital setting	Study period	Study population	Infection Type	Hospital wards	Bacteria
Fu et al. [44]	2014	case-control	matching/ significant test	Fujian	single	2012-2013	N/A	infection	N/A	*S. aureus*
Li et al. [15]	2016	retrospective	significant test	Guangdong	multiple	2008-2013	adult patients ≥18 years	N/A	N/A	*S. aureus*
Chen et al. [22]	2016	retrospective case-control	significant test	Jiangsu	single	2013-2014	adult patients ≥18 years	HAI pneumonia	N/A	*S. aureus*
			PSM/ significant test							
Hu et al. [42]	2014	retrospective	significant test	Zhejiang	multiple	2011-2012	N/A	NI	N/A	gram-negative
Long [20]	2015	retrospective case-control	matching/ significant test	Shanghai	single	2006-2014	children	BSI	N/A	gram-negative
Jiang et al. [40]	2016	retrospective	matching/ significant test	Shandong	single	2015	N/A	NI	N/A	gram-positive/ gram-negative
Li et al. [39]	2018	retrospective	significant test	Anhui	single	2016-2017	patients with bronchiectasis and infection	community-acquired infection	respiratory and ICU	gram-positive/ gram-negative
Liu [35]	2018	retrospective case-control	PSM/ significant test	Hubei	multiple	2013-2015	N/A			gram-positive/ gram-negative
Pan et al. [33]	2018	retrospective	significant test	Yunnan	single	2012-2015	surgical inpatients	infection	N/A	gram-positive/ gram-negative
Yang et al. [19]	2009	retrospective cohort			multiple	2005	N/A	N/A	N/A	gram positive/ gram negative

			multiple stepwise linear regression	Beijing/ Shandong/ Hubei/ Sichuan/ Ningxia						
Xing et al. [27]	2017	retrospective case-control	significant test	Shandong	single	2013-2015	N/A	infection	N/A	gram-positive/ gram-negative
Zhen et al. [5]	2018	retrospective	significant test	Zhejiang	single	2013-2015	N/A	IAI	N/A	gram-positive/ gram-negative
Guo et al. [43]	2017	retrospective cohort	PSM/ significant test	Shanghai	single	2008-2013	adult patients ⩾16 years	HAI	N/A	*A. baumannii*
Wu et al. [28]	2018	retrospective case-control	matching/ significant test	Chongqing	single	2014-2016	N/A	infection	N/A	*A. baumannii*
Cui et al. [21]	2012	retrospective case-control	matching/significant test	Beijing	single	2007-2009	N/A	NI	N/A	*A. baumannii*
Zhen et al. [4]	2017	retrospective	multivariate linear analysis	Zhejiang	single	2013-2015	N/A	N/A	N/A	*A. baumannii*
Chen et al. [6]	2018	retrospective cohort	significant test<br>PSM<br>significant test<br>PSM	Zhejiang	single	2014-2018	adult inpatients ⩾ 18 years	N/A	N/A	*P. aeruginosa*
Xu et al. [26]	2017	retrospective	significant test	Hubei	single	2015	N/A	infection	N/A	*E. coli*<br>*K. pneumonia*<br>*Proteus mirabilis*<br>*A. baumannii*<br>*P. aeruginosa*<br>*Enterobacter cloacae*<br>*S. aureus*<br>coagulase-negative *Staphylococci*
Hu et al. [2]	2010	retrospective	generalized linear model	Shanghai/ Beijing/ Zhejiang	multiple	2006-2007	N/A	IAI	N/A	*E. coli/ Klebsiella spp.*
Meng et al. [7]	2017	retrospective case-control	Significant test	Hunan	single	2012-2015	N/A	HAI	N/A	*E. coli*
Huang et al. [14]	2018	retrospective cohort	PAM/ significant test	Sichuan	single	2017	N/A	N/A	N/A	*K. pneumoniae*

PSM: propensity score matching; HAI: healthcare acquired infection; NI: nosocomial infection; BSI: bloodstream infection; IAI: intra-abdominal infection; ICU: intensive care unit; *A. baumannii*: *Acinetobacter baumannii*; *K. pneumoniae*: *Klebsiella pneumoniae*; *P. aeruginosa*: *Pseudomonas aeruginosa*; *S. aureus*: *Staphylococcus aureus*; *E. coli*: *Escherichia coli*.

Supplementary data 3: Study quality assessment

Table S4. Quality assessment checklist for nonrandomized studies.

Domain	Checklist Criteria	Additional Information
Selection	**1) Is the case definition adequate?**<br>a) yes, with independent validation *<br>b) yes, eg record linkage or based on self-reports<br>c) no description	
	**2) Representativeness of the cases**<br>a) consecutive or obviously representative series of cases *<br>b) potential for selection biases or not stated	Assumption: Given the nature of the review, studies received a star if they discuss representation (and reasons for their study being representative) or if they are multicentre/regional/national.
	**3) Selection of Controls**<br>a) community controls *<br>b) hospital controls<br>c) no description	Assumption: It was assumed that if the study was in a hospital setting in which cases were hospital patients, hospital controls were accepted.
	**4) Definition of Controls**<br>a) no history of disease (endpoint) *<br>b) no description of source	Assumption: History of disease/infection was used in this criteria even in studies looking at mortality or other burden outcomes
Comparability	**1) Comparability of cases and controls on the basis of the design or analysis**<br>a) study controls for age/sex/comorbidities *<br>b) study controls for any additional factor *	2 * maximum allotted for this criteria.<br>Assumption: For studies in which hospital associated cases and LoS were being analysed, two stars were only given if time dependency was controlled for
Exposure	**1) Ascertainment of exposure**<br>a) secure record (eg surgical records) *<br>b) structured interview where blind to case/control status *<br>c) interview not blinded to case/control status<br>d) written self-report or medical record only<br>e) no description	Assumption: Studies which utilised lab techniques were used to ascertain exposure received one star.
	**2) Same method of ascertainment for cases and controls**<br>a) yes *<br>b) no	
	**3) Non-Response rate**<br>a) same rate for both groups *<br>b) non respondents described<br>c) rate different and no designation	Assumption: No description of data cleaning or linkage and loss to missing data for retrospective studies was panelised by not awarding a star

Table S5. Study quality of the included study.

Study	Selection 1) *	Selection 2) *	Selection 3) *	Selection 4) *	Comparability 1) **	Exposure 1) *	Exposure 2) *	Exposure 3) *	Total (MAX. 9)
[1]	1	1	1	0	0	1	1	0	5
[2]	1	1	1	1	2	1	1	0	8
[3]	1	1	1	0	0	1	1	0	5
[4]	1	1	1	0	2	1	1	0	7
[5]	1	1	1	0	0	1	1	0	5
[6]	1	1	1	0	2	1	1	0	7
[7]	1	1	1	1	0	1	1	0	6
[8]	1	1	1	0	0	1	1	0	5
[9]	1	1	1	0	2	1	1	0	7
[10]	1	1	1	0	2	1	1	0	7
[11]	1	1	1	0	0	1	1	0	5
[12]	1	1	1	0	0	1	1	0	5
[13]	1	1	1	0	2	1	1	0	7
[14]	1	1	1	0	2	1	1	0	7
[15]	1	1	1	0	0	1	1	0	5
[16]	1	1	1	0	0	1	1	0	5
[17]	1	1	1	1	0	1	1	0	6
[18]	1	1	1	0	0	1	1	0	5
[19]	1	1	1	1	0	1	1	0	6
[20]	1	1	1	0	2	1	1	0	7
[21]	1	1	1	0	2	1	1	0	7
[22]	1	1	1	0	2	1	1	0	7

[23]	1	1	1	0	0	1	1	0	5
[24]	1	1	1	1	1	1	1	0	7
[25]	1	1	1	0	0	1	1	0	5
[26]	1	1	1	0	0	1	1	0	5
[27]	1	1	1	0	2	1	1	0	7
[28]	1	1	1	0	2	1	1	0	7
[29]	1	1	1	0	0	1	1	0	5
[30]	1	1	1	0	0	1	1	0	5
[31]	1	1	1	0	0	1	1	0	5
[32]	1	1	1	0	0	1	1	0	5
[33]	1	1	1	0	0	1	1	0	5
[34]	1	1	1	0	2	1	1	0	7
[35]	1	1	1	0	2	1	1	0	7
[36]	1	1	1	0	0	1	1	1	6
[37]	1	1	1	0	0	1	1	0	5
[38]	1	1	1	0	0	1	1	0	5
[39]	1	1	1	0	0	1	1	0	5
[40]	1	1	1	0	1	1	1	0	6
[41]	1	1	1	0	0	1	1	0	5
[42]	1	1	1	0	0	1	1	0	5
[43]	1	1	1	1	1	1	1	0	7
[44]	1	1	1	0	1	1	1	0	6

References

1. Zhu C, Liu C, Wu B, Wu Q, Huang D. Analysis of antibiotic resistance in the Staphylococcus aureus strains isolated from bloodstream infections and associated patient outcome. *Chinese Journal of Infection and Chemotherapy*. **2016**, *16*, 1-4.
2. Hu B, Ye H, Xu Y, Ni Y, Hu Y, Yu Y, Huang Z, Ma L. Clinical and economic outcomes associated with community-acquired intra-abdominal infections caused by extended spectrum beta-lactamase (ESBL) producing bacteria in China. *Curr. Med. Res. Opin.* **2010**, *26*, 1443-1449.
3. Yang S, Sun J, Wu X, Zhang L. Determinants of mortality in patients with nosocomial Acinetobacter baumannii bacteremia in southwest China: a five-year case-control study. *Can. J. Infect. Dis. Med.* **2018**, 1-9.
4. Zhen X, Chen Y, Hu X, Dong P, Gu S, Sheng YY, Dong H. The difference in medical costs between carbapenem-resistant Acinetobacter baumannii and non-resistant groups: a case study from a hospital in Zhejiang province, China. *Eur. J. Clin. Microbiol.* **2017**, *36*, 1989-1994.
5. Zhen X, Li Y, Chen Y, Dong P, Liu S, Dong H. Effect of multiple drug resistance on total medical costs among patients with intra-abdominal infections in China. *PLoS One*. **2018**, *13*.
6. Chen Z, Xu Z, Wu H, Chen L, Gao S, Chen Y. The impact of carbapenem-resistant Pseudomonas aeruginosa on clinical and economic outcomes in a Chinese tertiary care hospital: a propensity score-matched analysis. *Am. J. Infect. Control*. **2018**.
7. Meng X, Liu S, Duan J, Huang X, Zhou P, Xiong X, Gong R, Zhang Y, Liu Y, Fu C, Li C, Wu A. Risk factors and medical costs for healthcare-associated carbapenem-resistant Escherichia coli infection among hospitalized patients in a Chinese teaching hospital. *BMC Infect. Dis.* **2017**, *17*.
8. Zheng Y, Wan Y, Zhou L, Ye M, Liu S, Xu C, He Y, Chen J. Risk factors and mortality of patients with nosocomial carbapenem-resistant Acinetobacter baumannii pneumonia. *Am. J. Infect. Control*. **2013**, *41*, E59-E63.
9. Yuan L, Ding B, Shen Z, Wu H, Xu X, Li G. Clinical investigation of infections caused by carbapenem-resistant Pseudomonas aeruginosa in huashan hospital
. *Chinese Journal of Infection and Chemotherapy*. **2017**, *17*, 121-126.
10. Xiao T, Yu W, Niu T, Huang C, Xiao Y. A retrospective, comparative analysis of risk factors and outcomes in carbapenem-susceptible and carbapenem-nonsusceptible Klebsiella pneumoniae bloodstream infections: tigecycline significantly increases the mortality. *Infection and Drug Resistance*. **2018**, *11*, 595-606.
11. Wang Z, Qin R, Huang L, Sun L. Risk factors for carbapenem-resistant Klebsiella pneumoniae infection and mortality of Klebsiella pneumoniae infection. *Chinese Med. J.-Peking*. **2018**, *131*, 56-62.
12. Tian L, Tan R, Chen Y, Sun J, Liu J, Qu H, Wang X. Epidemiology of Klebsiella pneumoniae bloodstream infections in a teaching hospital: factors related to the carbapenem resistance and patient mortality. *Antimicrobial Resistance and Infection Control*. **2016**, *5*.
13. Jiao Y, Qin Y, Liu J, Li Q, Dong Y, Shang Y, Huang Y, Liu R. Risk factors for carbapenem-resistant Klebsiella pneumoniae infection/colonization and predictors of mortality: a retrospective study. *Pathog. Glob. Health*. **2015**, *109*, 68-74.
14. Huang W, Qiao F, Zhang Y, Huang J, Deng Y, Li J, Zong Z. In-hospital medical costs of infections caused by carbapenem-resistant Klebsiella pneumoniae. *Clin. Infect. Dis.* **2018**, *672*, S225-S230.
15. Li X, Chen Y, Gao W, Ouyang W, Wei J, Wen Z. Epidemiology and outcomes of complicated skin and soft tissue infections among inpatients in southern China from 2008 to 2013. *PLoS One*. **2016**, *11*.
16. Cao B, Wang H, Sun H, Zhu Y, Chen M. Risk factors and clinical outcomes of nosocomial multi-drug resistant Pseudomonas aeruginosa infections. *J. Hosp. Infect.* **2004**, *57*, 112-118.
17. Jia X, Ma W, Xu X, Yang S, Zhang L. Retrospective analysis of hospital-acquired linezolid-nonsusceptible enterococci infection in Chongqing, China, 2011-2014. *Am. J. Infect. Control*. **2015**, *43*, E101-E106.
18. Cai X, Sun J, Bao L, Li W. Risk factors and antibiotic resistance of pneumonia caused by multidrug resistant Acinetobacter baumannii in pediatric intensive care unit. *World Journal of Emergency Medicine*. **2012**, *3*, 202-207.
19. Yang L, Xiao Y, Wang J, Zheng Y, Yan Q, Hou F, Ma Y, Xu S, Sun Z, Lv X. Analysis of the effect of antibiotic resistance on hospital costs. *China Journal of Pharmaceutical Economics*. **2009**, 16-20.
20. Long Z. Clinical and economic impact of carbapenem resistance in Children's nonfermenters sepsis.: Shanghai Jiao Tong

University 2015: 72.
21. Cui N, Cao B, Liu Y, Liang L, Gu L, Song S. The impact of imipenem resistant Acinetobacter baumannii infection on clinical outcomes and medical care costs. *Chinese Journal of Infectious Diseases*. **2012**, *30*, 209-214.
22. Chen W, Li S, Li H, Zhang S, Liu B, Zhang X, Xu P, Zhang W. Comparison in prognosis of hospital-acquired pneumonia due to methicillin resistant and methicillin sensitive Staphylococcus aureus: analysis of propensity score matching. *Chinese Journal of Infection Control*. **2016**, *15*, 299-303.
23. Zhou L, Qiao F, Huang W, Zong Z. Impact of methicillin resistant Staphylococcus aureus bloodstream infection on the prognosis of patients. *Chinese Journal of Infection and Control*. **2015**, *14*, 27-30.
24. Zhang Z, Zhang J, Xie F, Lou G, Li X. Analysis of drug resistance of Staphyloccus aureus isolated from bloodstream and risk factors of methicillin resistant strains. *Laboratory Medicine and Clinic*. **2013**, *10*, 2380-2382.
25. Yu Y. Risk factors of nosocomial MRSA infection and molecular characteristics of Staphylococcus aureus isolated from blood.: Suzhou University 2016: 134.
26. Xu B, Yuan H, Yang P. Evaluation of economic burden induced by multi-drug resistant bacteria related infections in a tertiary general hospital. *Chinese Journal of Experimental and Clinical Infectious Diseases*. **2017**, *11*, 455-459.
27. Xing M, Qiu H, Wang L, Sun J. Targeted monitoring and economic burden evaluation on nosocomial infection of multi-drug resistant bacteria from 2013 to 2015. *Chinese Journal of Disinfection*. **2017**, *34*, 1045-1047.
28. Wu X, Ding L, Wu X. Direct economic loss due to healthcare-associated infection with multi-drug resistant Acinetobacter baumannii. *Chinese Journal of Infection Control*. **2018**, *17*, 735-738.
29. Wang Y, Fu R, Zheng Y, Wan Y, Zhou L. Risk factors and mortality of patients with nosocomial carbapenem-resistant Acinetobacter baumannii pneumonia. *Journal of Clinical Pulmonary Medicine*. **2016**, *21*, 784-788.
30. Jiang D, Zuo S, Liang Y, Li H. Risk factor for multi-drug resistant organism infection in department of respiratory medicine. *Journal of North Sichuan Medical College*. **2016**, *31*, 730-733.
31. Wang D. Clinical characteristics and risk factors of carbapenem resistant gram-negative bacteria bloodstream infection.: Zhejiang University 2018: 45.
32. Pei H. Clinical characteristics, risk factors for occurrence and prognosis analysis for multidrug-resistant Acinetobacter baumannii in a tertiary care hospital from 2010 to 2012.: Anhui Medical University 2015: 51.
33. Pan H, Chen Q, Chu C, Chen L, Yang B, Liang C. Risk factors of multi-drug resistant infection in surgical sites. *Practical Preventive Medicine*. **2018**, *25*, 367-369.
34. Lv Q, Ruan Z, Wang J, Ma Q, Dai Y. Clinical characteristics and prognosis of patients with carbapenem resistant Pseudomonas aeruginosa infection. *Chinese Journal of Nosocomiology*. **2015**, *25*, 5570-5571.
35. Liu X. Estimating the direct economic disease burden of healthcare associated infections with antimicrobial resistance (AMR) of tertiary hospital in Hubei province.: Wuhan University 2018: 69.
36. Liang L. Mechanical ventilation in patients with multi-drug resistant (MDR) Acinetobacter infection distribution characteristics and drug resistance affecting factors.: Hebei United University 2014: 48.
37. Li H, Lin X, Hua Z, Tu X, Yu M, Hu X, Yao X, Chen Y. Risk factors for infection with Klebsiella pneumoniae producing drug-resistant enzymes in respiratory department. *Chinese Journal of Clinical Research*. **2016**, *29*, 185-188.
38. Li H, Sun S, Zhou H. Analysis of clinical features and outcomes of infections with multi-drug resistant organisms in certain intensive care unit in 2011. *Chinese Journal of Nosocomiology*. **2014**, *24*, 1643-1644.
39. Li C, Han M, Zhou X, Huang J. Study on the distribution of pathogenic bacteria and the direct economic burden caused by drug resistance in patients with bronchiectasis and infection. *Journal of Clinical Pulmonary Medicine*. **2018**, *23*, 1680-1683.
40. Jiang X, Sun J, Xing M, Wang L, Zhang X, Qiu H. Characteristics and the direct economic loss of multi-drug resistant organisms nosocomial infection in a general hospital. *Chinese Journal of Nosocomiology*. **2016**, *26*, 5727-5729.
41. Huang J. Risk factors and clinical outcomes of carbapenem-resistant Klebsiella pneumoniae infections of critically ill patients.: Zhejiang University 2015: 44.
42. Hu G, Shen L, Jiang S, Chen W, Wang Z. Impact of multi-drug gram-negative bacilli bloodstream infection on outcome of patients. *Zhejiang Medical Journal*. **2014**, *36*, 1942-1944.
43. Guo Y, Guo W, Qiu P, He Q, Pan C, Wu P, He J. Study of attributive hospitalized cost and length of stay for hospital acquired

infection due to multidrug resistance and non-resistance Acinetobacter baumannii. *Chinese Journal of Health Statistics*. **2017**, *34*, 378-381.

44. Fu J, Baoqin C, Wang X, Ye Q, Lu Z, Su C, Niu J. Case-control study of influence of methicillin-resistant Staphylococcus aureus infection on economic losses and length of hospital stay. *Chinese Journal of Nosocomiology*. **2014**, *24*, 2363-2365.
